# Clinical utility of self-reported sleep duration and insomnia symptoms in type 2 diabetes prediction

**DOI:** 10.1007/s00125-025-06503-6

**Published:** 2025-08-02

**Authors:** Alison K. Wright, Tianyi Huang, Matthew J. Carr, Arjun D. Premdayal, Sushant Saluja, Hassan S. Dashti, Simon G. Anderson, David W. Ray, Samuel E. Jones, Andrew R. Wood, Timothy M. Frayling, Michael N. Weedon, Jacqueline M. Lane, Richa Saxena, Junxi Liu, Jack Bowden, Deborah A. Lawlor, Susan Redline, Martin K. Rutter

**Affiliations:** 1 Division of Diabetes, Endocrinology and Gastroenterology, School of Medical Sciences, University of Manchester, Manchester, UK; 2 Centre for Pharmacoepidemiology and Drug Safety, Division of Pharmacy and Optometry, School of Health Sciences, University of Manchester, Manchester Academic Health Sciences Centre, Manchester, UK; 3 Channing Division of Network Medicine, Brigham and Women’s Hospital, Boston, MA USA; 4 Harvard Medical School, Boston, MA USA; 5 Royal Liverpool University Hospital, Liverpool, UK; 6 Division of Cardiovascular Sciences, University of Manchester, Manchester, UK; 7 Department of Anesthesia, Critical Care and Pain Medicine, Massachusetts General Hospital and Harvard Medical School, Boston, MA USA; 8 Chronic Disease Research Centre of CAIHR, The University of the West Indies, Bridgetown, Barbados; 9 NIHR Oxford Health Biomedical Research Centre, John Radcliffe Hospital, Oxford, UK; 10 Oxford Centre for Diabetes, Endocrinology and Metabolism, University of Oxford, Oxford, UK; 11 Oxford Kavli Centre for Nanoscience Discovery, University of Oxford, Oxford, UK; 12 Institute for Molecular Medicine, HiLIFE, University of Helsinki, Helsinki, Finland; 13 Genetics of Complex Traits, College of Medicine and Health, University of Exeter, Exeter, UK; 14 Department of Genetic Medicine and Development, CMU, University of Geneva, Geneva, Switzerland; 15 Division of Sleep and Circadian Disorders, Brigham and Women’s Hospital, Boston, MA USA; 16 Center for Genomic Medicine, Massachusetts General Hospital, Boston, MA USA; 17 Nuffield Department of Population Health, Oxford Population Health, University of Oxford, Oxford, UK; 18 MRC Integrative Epidemiology Unit, University of Bristol, Bristol, UK; 19 Population Health Sciences, Bristol Medical School, University of Bristol, Bristol, UK; 20 College of Medicine & Health, University of Exeter, Exeter, UK; 21 Beth Israel Deaconess Medical Center, Boston, MA USA; 22 Diabetes, Endocrinology and Metabolism Centre, Manchester University NHS Foundation Trust, NIHR Manchester Biomedical Research Centre, Manchester Academic Health Sciences Centre, Manchester, UK

**Keywords:** Insomnia, Prediction, Risk assessment, Sleep deprivation, Type 2 diabetes

## Abstract

**Aims/hypothesis:**

Suboptimal sleep health is linked to higher risks for incident type 2 diabetes. We aimed to assess the clinical utility of adding self-reported sleep traits to a type 2 diabetes prediction model.

**Methods:**

In this cohort study, we used UK Biobank data and Cox proportional hazards models to examine how self-reported sleep duration and insomnia symptoms were associated with incident type 2 diabetes risk. Harrell’s C statistic and net reclassification improvement (NRI) were used to assess whether sleep traits improved the incident type 2 diabetes discrimination and predictive utility achieved using QDiabetes variables, with and without including a type 2 diabetes polygenic risk score (PGS). Independent replication was explored in the Nurses’ Health Study, the Nurses’ Health Study II and the Health Professionals Follow-up Study.

**Results:**

Extremes of sleep duration and occasional or frequent insomnia symptoms were associated with higher risks for incident type 2 diabetes. In the UK Biobank and replication cohorts, adding sleep traits to the QDiabetes risk score did not improve type 2 diabetes prediction (C statistic: QDiabetes alone 0.8933; QDiabetes + sleep duration 0.8939; QDiabetes + insomnia 0.8931; QDiabetes + sleep traits 0.8935). The corresponding total NRI values were: 0.08 (95% CI −0.18, 0.33), 0.04 (95% CI −0.08, 0.16) and 0.04 (95% CI −0.10, 0.18). Inclusion of PGS data marginally improved the type 2 diabetes risk prediction achieved using The QDiabetes calculator, with or without the inclusion of sleep traits in the model (QDiabetes + PGS: C statistic 0.8945; total NRI 0.20 [95% CI 0.12, 0.28]; QDiabetes + PGS + sleep traits: C statistic 0.8946; total NRI 0.18 [95% CI 0.09, 0.27]).

**Conclusions/interpretation:**

While sleep duration and insomnia symptoms were associated with type 2 diabetes risk, they are not useful for improving type 2 diabetes prediction beyond QDiabetes model performance. Inclusion of a type 2 diabetes PGS marginally improved prediction but lacked clear clinical utility.

**Graphical Abstract:**

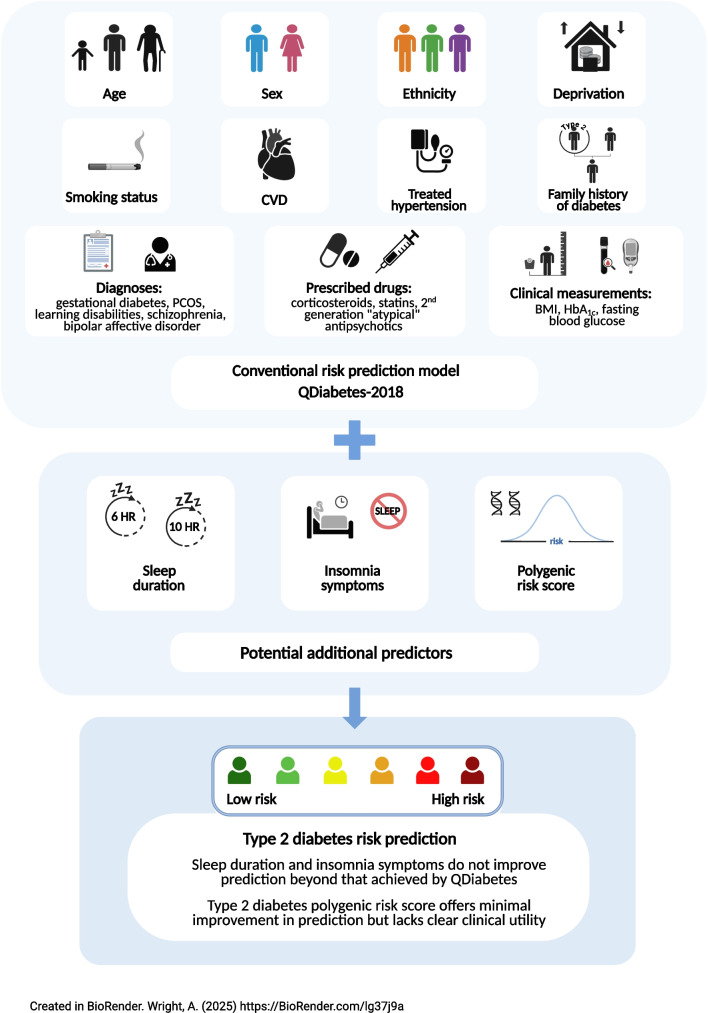

**Supplementary Information:**

The online version of this article (10.1007/s00125-025-06503-6) contains peer-reviewed but unedited supplementary material.


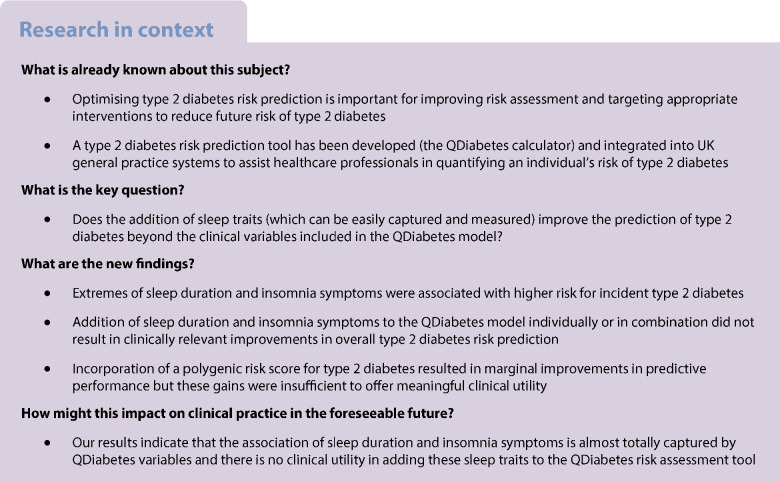


## Introduction

Optimising type 2 diabetes prediction is critical for prevention strategies. In the UK, the QDiabetes prediction tool (https://qdiabetes.org/) helps primary care clinicians to identify people at risk from type 2 diabetes using 17 variables (including age, ethnicity and body weight [1]) to provide a high level of prediction (Harrell’s C statistic of approximately 0.87) [2].

Extremes of sleep duration and insomnia are known to predict type 2 diabetes [3], but no study has assessed whether including sleep traits improves prediction beyond the QDiabetes model. While type 2 diabetes polygenic risk scores (PGS) modestly improve prediction when added to conventional models [4], no study has assessed how adding sleep traits compares to adding PGS data to the QDiabetes model.

Therefore, our aims were: (1) to assess whether sleep duration and/or insomnia symptoms improve type 2 diabetes prediction beyond that achieved using the QDiabetes calculator; (2) to compare models with and without PGS; and (3) to replicate the findings in independent cohorts. In secondary analyses, we repeated our main analyses excluding BMI, fasting blood glucose and HbA_1c_ to assess predictive performance using only person-reported factors, which may be helpful outside clinical settings, and also repeated the main analyses in people with depression whose type 2 diabetes risk may be particularly affected by suboptimal sleep [5, 6].

## Methods

### Study participants

We used data on 502,540 UK Biobank (UKB) participants aged 40–69 years at recruitment (2006–2010) (see electronic supplementary material [ESM] Methods) [7]. Briefly, all participants provided questionnaire data on sleep and prevalent diabetes, had BMI assessed and had a blood sample taken for determination of HbA_1c_, glucose and genotype [7]. We excluded participants with prevalent diabetes [8], obstructive sleep apnoea, use of sleep-modifying medication, missing data on self-reported sleep traits and type 1 diabetes diagnosed after enrolment and those who later withdrew consent, leaving 492,114 participants.

The replication cohort comprised data from three US prospective cohort studies: the Nurses’ Health Study (NHS; comprising 121,700 female registered nurses aged 30–55 years enrolled in 1976) [9], the Nurses’ Health Study II (NHSII; 116,430 female registered nurses aged 25–42 years at baseline in 1989) [9] and the Health Professionals Follow-up Study (HPFS; 51,529 male health professionals aged 40–75 years at baseline in 1986) [10, 11]. All participants completed a baseline questionnaire, and were invited to complete lifestyle, health and medication questionnaires every 2 years [9–11]. Blood samples were collected in subsets of participants [9, 12]. For the current analysis, follow-up started in 2000 (NHS/HPFS) and 2001 (NHSII), when sleep traits were consistently assessed. We excluded participants with diabetes diagnosed at follow-up commencement or with incomplete sleep trait data, leaving 183,920 participants (74,277 women from the NHS, 83,061 women from the NHSII and 26,582 men from the HPFS).

The questionnaires used in the UKB, NHS/NHSII and HPFS can be accessed at https://biobank.ndph.ox.ac.uk/showcase/docs.cgi?id=0 (UKB), https://nurseshealthstudy.org/participants/questionnaires (NHS/NHSII) and https://hsph.harvard.edu/research/health-professionals/questionnaires/ (HPFS).

### Sleep exposures

In the UKB cohort, we considered baseline sleep duration and insomnia symptoms as exposures. Sleep duration was self-reported as the number of hours of sleep generally obtained in every 24 h, including naps (inputted as an integer value between 1 and 23 hours). We categorised participants into six groups: ≤5 h, 6 h, 7 h (referent group), 8 h, 9 h and ≥10 h. For insomnia symptoms, participants were asked: ‘In relation to the last 4 weeks, do you have trouble falling asleep at night or do you wake up in the middle of the night’, with the response options ‘usually’, ‘sometimes’, ‘never/rarely’ (referent group) and ‘prefer not to answer’.

In the validation cohorts, sleep duration over 24 h was self-reported using categories (<5 h, 5 h, 6 h, 7 h, 8 h, 9 h, 10+ h), although this question did not include naps. The responses <5 h and 5 h were combined to create a category ≤5 h to correspond with the UKB. Insomnia symptoms were assessed by the question: ‘Did you have difficulty falling asleep or staying asleep during the past 4 weeks?’. To be consistent with the UKB cohort, participants who reported ‘all of the time’ or ‘most of the time’ were considered as ‘usually’ having symptoms; those who reported ‘a good bit of the time’ or ‘some of the time’ were considered as ‘sometimes’ having symptoms; and those who reported ‘a little of the time’ or ‘none of the time’ were considered as ‘never/rarely’ having symptoms.

### Outcome

In the UKB cohort, incident diabetes was defined as the earliest occurrence of type 2 diabetes from the Hospital Episodes Statistics dataset, GP records (when available) or self-reported physician diagnosis at follow-up assessments.

In the NHS/NHSII/HPFS cohorts, incident diabetes was self-reported on biennial questionnaires; positive respondents were sent a supplementary questionnaire on symptoms, diagnostic tests and treatment. Self-reported diabetes was validated against ADA diagnostic criteria [13–15].

### QDiabetes variables and PGS

QDiabetes risk factors included in this study were age, sex, ethnicity (in UKB defined as White, Asian, Black, Chinese, mixed, other, unknown and in NHS/NHSII/HPFS defined as White, Asian, Black, Mixed/other/unknown), Townsend deprivation score [16], BMI, fasting blood glucose, HbA_1c_, smoking status, family history of diabetes, hypertension, CVD, schizophrenia or bipolar affective disorder, gestational diabetes, polycystic ovary syndrome (PCOS) and use of corticosteroids, second-generation antipsychotics and/or statins. Across the cohorts, ethnicity was categorised into four groups; White, Asian, Black and Mixed/other/unknown. The following data was not collected in NHS/NHSII/HPFS: Townsend deprivation score, fasting blood glucose, diagnoses of schizophrenia, bipolar affective disorder or PCOS (only available in NHSII) and use of second-generation antipsychotics. As baseline HbA_1c_ was available for only a small subset of NHS/NHSII/HPFS participants (Table 1), this was not included in the analysis of these cohorts.

**Table 1:** Baseline characteristics of UKB participants and validation cohorts (NHS/NHSII/HPFS)

	UKB cohort (*N *= 492,114)	Validation cohorts (*N *= 183,920)
Age, years	56.5 ± 8.1	57.5 ± 11.2
Sex, male	223,446 (45.4)	26,582 (14.5)
Ethnicity		
White	464,451 (94.4)	172,987 (94.1)
Asian	7780 (1.6)	1977 (1.1)
Black	7681 (1.6)	1984 (1.1)
Mixed, other or unknown	12,202 (2.5)	6972 (3.8)
Townsend deprivation score quintiles^a^		
1 (−6.26 to −3.94)	98,409 (20.0)	
2 (−3.94 to −2.78)	98,213 (20.0)	
3 (−2.78 to −1.33)	98,285 (20.0)	
4 (−1.33 to 1.29)	98,304 (20.0)	
5 (1.29 to 11.00)	98,297 (20.0)	
Unknown	606 (0.1)	
Shift worker		
Never/rarely	234,101 (47.6)	
Sometimes	20,311 (4.1)	
Usually or always	28,290 (5.8)	
Unknown	209,412 (42.6)	
BMI, kg/m^2^	27.4 ± 4.7	26.5 ± 5.4
Missing	2725 (0.6)	
Fasting blood glucose, mmol/l	5.1 ± 1.2	
Missing	68,791 (14.0)	
HbA_1c_, mmol/mol	36 ± 6	37 ± 4
HbA_1c_, %	5.4 ± 0.6	5.5 ± 0.4
Missing	33,439 (6.8)	174,575 (94.9)
Type 2 diabetes PGS^b^	–4.0 × 10^–11^ ± 1.00	−0.03 ± 1.00
Missing	14,486 (2.9)	148,848 (80.9)
Smoking status		
Current smoker	51,655 (10.5)	15,122 (8.2)
Former smoker	113,077 (23.0)	67,762 (36.8)
Non-smoker	327,382 (66.5)	101,036 (54.9)
Family history of diabetes	105,901 (21.5)	54,924 (29.9)
Treated hypertension	100,803 (20.5)	43,196 (23.5)
History of CVD	28,340 (5.8)	9820 (5.3)
Schizophrenia or bipolar disorders	1928 (0.4)	
Depression	27,132 (5.5)	28,495 (15.5)
Gestational diabetes	783 (0.2)	722 (0.4)
Polycystic ovary syndrome^c^	612 (0.1)	2,917 (3.5)
Corticosteroids	1921 (0.4)	3684 (2.0)
Second-generation antipsychotics	803 (0.2)	
Statins	78,969 (16.0)	25,229 (13.7)
Chronotype^c^		
Definitely ‘morning’ person	118,398 (24.1)	23,533 (28.3)
More ‘morning’ than ‘evening’	155,166 (31.5)	17,537 (21.1)
More ‘evening’ than ‘morning’	124,100 (25.2)	15,037 (18.1)
Definitely ‘evening’ person	39,108 (8.0)	7789 (9.4)
Neither		3407 (4.1)
Unknown	55,342 (11.2)	15,758 (19.0)
Sleep duration, h/day		
≤5	27,044 (5.5)	9723 (5.3)
6	94,278 (19.2)	41,800 (22.7)
7	190,409 (38.7)	74,002 (40.2)
8	142,681 (29.0)	46,290 (25.2)
9	28,730 (5.8)	10,083 (5.5)
≥10	8972 (1.8)	2022 (1.1)
Insomnia symptoms		
Never/rarely	119,126 (24.2)	124,175 (67.5)
Sometimes	235,398 (47.8)	49,539 (26.9)
Usually	137,590 (28.0)	10,206 (5.5)

Values for continuous variables are means ± SD; values for categorical variables are *n* (%)

^a^For the Townsend deprivation score, quintile 1 indicates least deprived; quintile 5 indicates most deprived; the score range is provided for each quintile

^b^Standardised to *z* score (in individuals with sleep data in the UKB cohort and in validation cohorts, a larger cohort with genotyping data). The PGS was calculated based on 110 SNPs in the UKB cohort and 116 SNPs in the NHS/NHSII/HPFS cohorts (out of 128 SNPs reported to be associated with type 2 diabetes in prior genome-wide association studies)

^c^In validation cohorts, PCOS and chronotype information was only available in NHSII (*N *= 83,061)

A PGS for type 2 diabetes, as described previously [17], was calculated based on 110 SNPs in the UKB cohort and 116 SNPs in NHS/NHSII/HPFS cohorts (out of 128 SNPs reported to be associated with type 2 diabetes in prior genome-wide association studies). In NHS/NHSII/HPFS cohorts there was a high proportion of missing data for PGS (80.9%).

### Statistical analysis

Baseline characteristics were summarised using means ± SD or proportions as appropriate. Crude type 2 diabetes incidence rates (per 1000 person-years) were calculated for each category of sleep duration and insomnia symptoms.

In the UKB cohort, we used multiple imputation with chained equations (MICE) to derive missing values for BMI (0.6%), fasting blood glucose (14%), HbA_1c_ (6.8%), deprivation (0.1%) and PGS (2.9%); 19.3% of participants had missing data for at least one variable. Thirty complete datasets were imputed using all sleep traits and time to outcome. In the NHS/NHSII/HPFS cohorts, data from the prior questionnaire cycle were carried forward to impute missing BMI and smoking status (<3% missingness). Cox proportional hazard regression models were used to estimate associations of sleep duration and insomnia symptoms with incident type 2 diabetes risk. Participants were observed from baseline until type 2 diabetes diagnosis date, death or the end of available follow-up (May 2015 for UKB participants, June 2016 for the NHS, June 2017 for the NHSII and January 2016 for HPFS), whichever occurred first. Model adjustments included QDiabetes risk factors and a type 2 diabetes PGS. Age, BMI, blood glucose and HbA_1c_ were mean-centred, and PGS was standardised. Proportionality was assessed using Schoenfeld residuals. The performance of QDiabetes prediction models including sleep duration, insomnia symptoms and/or PGS was compared with the performance of the standard QDiabetes model using: (1) Harrell’s C statistic, a measure of the ability of the model to discriminate between those who develop type 2 diabetes and those who do not [18]; and (2) net reclassification improvement (NRI), a comparison of two risk models constructed with and without sleep traits/PGS to quantify whether sleep traits/PGS provide clinically relevant prediction improvements [19, 20]. The event NRI is the net proportion of events assigned a higher risk, while the non-event NRI is the net proportion of non-events assigned a lower risk. NRI values range from −2 to 2, with negative values indicating that the new markers result in a net worsening in risk classification [20, 21]. Likelihood ratio test *p* values were calculated to assess whether significant improvements in the Cox model data fit were observed with the inclusion of sleep duration, insomnia symptoms and/or PGS compared with the standard QDiabetes model.

Secondary analyses excluded BMI, fasting blood glucose and HbA_1c_ to compare the predictive abilities achieved using easy-to-measure person-reported risk factors, which could be helpful outside clinical settings. We also repeated analyses in people with depression (self-reported illness in the UKB cohort, self-reported physician-diagnosed or antidepressant use in validation cohorts), as sleep disturbance may have a particularly important role in type 2 diabetes in these individuals [22]. All analyses were replicated in US cohorts. Analysis was performed using Stata 16.1 (StataCorp, USA).

## Results

### Baseline characteristics

The mean age (± SD) of participants at baseline was 56.5 ± 8.1 years, 223,446 (45.4%) were male, 464,451 (94.4%) self-reported their ethnicity as White, 164,732 (33.5%) were current or former smokers and 105,901 (21.5%) had a family history of diabetes (Table 1). Their fasting plasma glucose was 5.1 ± 1.2 mmol/l, HbA_1c_ was 36 ± 6 mmol/mol (5.4±0.6%) and BMI was 27.4±4.7 kg/m^2^ (means ± SD). Most participants reported sleeping 7–8 h per day (68%), with 5.5% sleeping for ≤5 h per day and 1.8% sleeping for ≥10 h per day. Insomnia symptoms were experienced by 28% of participants frequently (‘usually’ in the questionnaire response).

Across the three replication cohorts (NHS/NHSII/HPFS), 183,920 people were included (Table 1), with a mean age (± SD) of 57.5±11.2 years; 26,582 (14.5%) were male; 172,987 (94.1%) self-reported their ethnicity as White; 82,884 (45.1%) were current or former smokers; and 54,924 (29.9%) had a family history of diabetes. Their HbA_1c_ was 37±4 mmol/mol (5.5%±0.4%) and BMI was 26.5±5.4 kg/m^2^ (means ± SD). Most reported sleeping 7–8 h per day (65%), with similar proportions to UKB participants reporting extremes of sleep duration (≤5 h/day 5.3%, ≥10 h/day 1.1%), but only 6% reported having insomnia symptoms regularly (‘all of the time’ or ‘most of the time’ in the questionnaire response).

### Incident type 2 diabetes

In UKB participants, we identified 8092 incident type 2 diabetes cases, arising from over 3 million person-years of observation (incidence rate/1000 person-years 2.65; 95% CI 2.59, 2.71) (Table 2). In the NHS/NHSII/HPFS cohorts, 12,197 incident type 2 diabetes cases were identified over 2.6 million person-years of observation (incidence rate/1000 person-years 4.64; 95% CI 4.56, 4.72).

**Table 2:** Incidence (with 95% CI) for type 2 diabetes per 1000 person-years by baseline sleep trait status

	UKB cohort (*N*=492,114)			Validation cohorts (*N *= 183,920)		
	Type 2 diabetes	Person-years	Incidence^a^	Type 2 diabetes	Person-years	Incidence^a^
*N*	8092	3,056,637	2.65 (2.59, 2.71)	12,197	2,628,319	4.64 (4.56, 4.72)
Sleep duration, h/day						
≤5	708	166,337	4.26 (3.95, 4.58)	874	134,949	6.48 (6.06, 6.92)
6	1750	583,673	3.00 (2.86, 3.14)	3076	598,492	5.14 (4.96, 5.32)
7	2511	1,187,349	2.11 (2.03, 2.20)	4545	1,073,709	4.23 (4.11, 4.36)
8	2153	887,624	2.43 (2.33, 2.53)	2843	658,404	4.32 (4.16, 4.48)
9	637	177,304	3.59 (3.32, 3.88)	698	138,071	5.06 (4.69, 5.44)
≥10	333	54,350	6.13 (5.50, 6.82)	161	24,695	6.52 (5.59, 7.61)
Insomnia symptoms						
Never/rarely	1579	741,399	2.13 (2.03, 2.24)	7641	1,791,193	4.27 (4.17, 4.36)
Sometimes	3597	1,464,356	2.46 (2.38, 2.54)	3609	697,271	5.18 (5.01, 5.35)
Usually	2916	850,883	3.43 (3.30, 3.55)	947	139,856	6.77 (6.35, 7.22)

^a^Per 1000 person-years

### Predictor variables

**Fig. 1:**
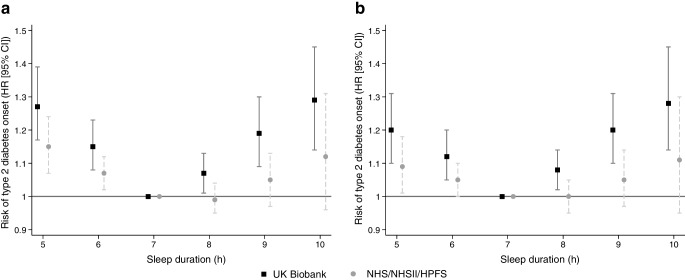
Relationship of sleep duration with risk (HR and 95% CI) for incident type 2 diabetes (7 h/day as referent group) after (**a**) adjusting for QDiabetes variables and (**b**) after adjusting for QDiabetes variables plus insomnia symptoms, in the UKB and validation cohorts (NHS/NHSII/HPFS). The QDiabetes model includes age, sex, ethnicity, Townsend deprivation score, BMI, fasting blood glucose, HbA_1c_, smoking status, family history of diabetes, hypertension, CVD, schizophrenia or bipolar affective disorder, gestational diabetes PCOS and use of corticosteroids, second-generation antipsychotics and/or statins. In the NHS/NHSII/HPFS cohorts, the QDiabetes model did not include fasting blood glucose, HbA_1c_, Townsend deprivation score, diagnoses of schizophrenia, bipolar affective disorder or PCOS and use of second-generation antipsychotics

In the UKB cohort, after adjusting for QDiabetes variables (Fig. 1a), a U-shaped relationship was observed between sleep duration and type 2 diabetes risk, with the greatest risks observed at extremes of sleep duration. After adjusting for QDiabetes variables and insomnia symptoms (Fig. 1b), type 2 diabetes risk was 20% higher in those sleeping ≤5 h per day (HR 1.20; 95% CI 1.10, 1.31) and 28% higher in those sleeping ≥10 h per day (HR 1.28; 95% CI 1.13, 1.44), compared with those sleeping 7 h/day. Similar U-shaped associations were observed in the NHS/NHSII/HPFS cohorts, although with lower risk estimates at all sleep durations.

**Fig. 2:**
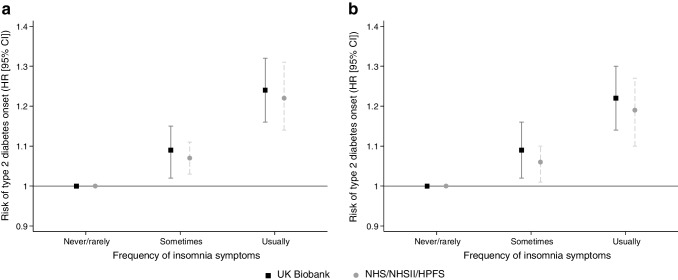
Relationship of insomnia symptoms with risk (HR and 95% CI) for incident type 2 diabetes (‘never/rarely’ as referent group) after adjusting for (**a**) QDiabetes variables, and (**b**) after adjusting for QDiabetes variables plus sleep duration, in the UKB and validation cohorts (NHS/NHSII/HPFS). The QDiabetes model includes age, sex, ethnicity, Townsend deprivation score, BMI, fasting blood glucose, HbA_1c_, smoking status, family history of diabetes, hypertension, CVD, schizophrenia or bipolar affective disorder, gestational diabetes, PCOS and use of corticosteroids, second-generation antipsychotics and/or statins. In the NHS/NHSII/HPFS cohorts, the QDiabetes model did not include fasting blood glucose, HbA_1c_, Townsend deprivation score, diagnoses of schizophrenia, bipolar affective disorder or PCOS and use of second-generation antipsychotics

After adjusting for QDiabetes variables and sleep duration, similar associations between insomnia symptoms and type 2 diabetes risk were evident in both UKB and NHS/NHSII/HPFS participants (Fig. 2b). People reporting experiencing insomnia symptoms ‘sometimes’ or ‘usually’ had higher risks of developing type 2 diabetes than those never or rarely experiencing insomnia symptoms: HR 1.09 (95% CI 1.03, 1.16) and HR 1.22 (95% CI 1.14, 1.30), respectively. The corresponding HRs (95% CI) in NHS/NHSII/HPFS participants were 1.05 (1.01, 1.10) and 1.18 (1.10, 1.26), respectively.

### Assessment of prediction models

Table 3 compares type 2 diabetes prediction, assessed by C statistics and NRI, using the QDiabetes model with and without the addition of sleep duration and/or insomnia symptoms, in UKB and NHS/NHSII/HPFS participants.

**Table 3:** Comparison of type 2 diabetes prediction, assessed by C statistics and NRI, using the QDiabetes calculator with and without self-reported sleep duration and insomnia symptoms, in the UKB and validation cohorts

	Harrell’s C statistic	NRI			Data fit (*p* value)^a^
		Event NRI	Non-event NRI	Total NRI	
UKB cohort					
QDiabetes	0.8933				
QDiabetes + sleep duration	0.8939	−0.16 (−0.39, 0.07)	0.24 (−0.16, 0.64)	0.08 (−0.18, 0.33)	0.53
QDiabetes + insomnia symptoms	0.8931	−0.32 (−0.61, −0.03)	0.36 (0.03, 0.69)	0.04 (−0.08, 0.16)	0.77
QDiabetes + PGS	0.8945	0.07 (−0.02, 0.15)	0.14 (0.13, 0.15)	0.20 (0.12, 0.28)	<0.001
QDiabetes + sleep duration + insomnia symptoms	0.8935	−0.04 (−0.17, 0.10)	0.08 (−0.07, 0.23)	0.04 (−0.10, 0.18)	0.66
QDiabetes + sleep duration + insomnia symptoms + PGS	0.8946	0.04 (−0.05, 0.13)	0.14 (0.12, 0.15)	0.18 (0.09, 0.27)	0.001
Validation cohorts^b^					
QDiabetes	0.8040				
QDiabetes + sleep duration	0.8043	−0.27 (−0.42, −0.13)	0.35 (0.20, 0.49)	0.07 (−0.02, 0.17)	0.76
QDiabetes + insomnia symptoms	0.8041	−0.27 (−0.35, −0.19)	0.35 (0.34, 0.37)	0.08 (0.01, 0.16)	0.79
QDiabetes + PGS^c^	0.8193	0.20 (0.07, 0.32)	0.13 (0.12, 0.15)	0.33 (0.20, 0.46)	<0.001
QDiabetes + sleep duration + insomnia symptoms	0.8043	−0.17 (−0.38, 0.04)	0.13 (−0.05, 0.32)	−0.04 (−0.15, 0.07)	0.91
QDiabetes + sleep duration + insomnia symptoms + PGS^c^	0.8209	0.18 (0.04, 0.32)	0.14 (0.12, 0.16)	0.32 (0.18, 0.46)	<0.001

Values for the C statistics are AUCs; values for NRI are score (95% CI)

^a^*p* values were calculated using the Cox model likelihood ratio test to assess whether significant improvements in the Cox model data fit were observed with the inclusion of sleep duration, insomnia symptoms and/or PGS compared with the standard QDiabetes model

^b^The QDiabetes model in the validation cohorts (NHS/NHSII/HPFS) did not include fasting blood glucose, HbA_1c_, Townsend deprivation score, diagnoses of schizophrenia, bipolar affective disorder or PCOS and use of second-generation antipsychotics

^c^Based on 35,072 NHS/NHSII/HPFS participants with genetic data (the Harrell’s C statistic for the QDiabetes base model was 0.8565 in this subset)

In UKB participants, the QDiabetes model performed well in terms of event discrimination, with a C statistic for predicting incident type 2 diabetes of 0.8933 (Table 3). Addition of sleep duration and insomnia symptoms individually did not change the C statistic. In general, addition of sleep traits (duration and/or insomnia symptoms) individually or in combination with the QDiabetes model led to reductions in sensitivity (negative event NRIs) but improved specificity (positive non-event NRIs), demonstrating the model’s ability to correctly classify individuals who do not develop type 2 diabetes, with non-significant improvements in overall reclassification (total NRI).

Incident type 2 diabetes discrimination using the QDiabetes model was marginally improved through adding the diabetes PGS alone (C statistic 0.8945) or PGS together with sleep duration and insomnia symptoms (C statistic 0.8946). Adding the PGS to the QDiabetes base model led to no change in sensitivity (event NRI 0.07 [95% CI −0.02, 0.15]) but led to statistically significant but small improvements in both specificity (non-event NRI 0.14 [0.13, 0.15]) and total NRI (0.20 [0.12, 0.28]). Similarly, when the PGS was added to the QDiabetes base model together with both sleep traits; there was no change in sensitivity (event NRI 0.04 [−0.05, 0.13]) but statistically significant small improvements in non-event NRI (0.14 [0.12, 0.15]) and total NRI (0.18 [0.09, 0.27]).

While similar results were found when stratified by sex (ESM Table 1), the QDiabetes model provided marginally greater discrimination in female participants than male participants (0.9003 vs 0.8851, respectively). In female participants, discrimination using the QDiabetes base model improved moderately with addition of the PGS (C statistic 0.9018) but not with addition of sleep traits individually or in combination. Inclusion of sleep traits (duration and insomnia symptoms) individually to the QDiabetes model led to reductions in sensitivity (negative event NRIs) but improved specificity (positive non-event NRIs), with non-significant improvements in event, non-event and total NRI when both sleep traits were included. In male participants, adding sleep duration alone provided minimal improvements in discrimination (C statistic 0.8872) but adding insomnia symptoms or PGS, individually or in combination, did not. Minimal but statistically significant improvements in specificity and overall classification only occurred when sleep traits and PGS were included together.

Discrimination using the QDiabetes model differed by ethnicity (ESM Table 2), with C statistics of 0.8968 for White, 0.8591 for South Asian, 0.9641 for African Caribbean and 0.8568 for mixed/other ethnic groups. For South Asian, African Caribbean and mixed/other ethnic groups, while there were changes in sensitivity and specificity with the addition of sleep traits and PGS, individually and in combination, these improvements were marginal when compared with QDiabetes prediction alone, which was exceptionally good, especially in African Caribbeans (ESM Table 2).

The QDiabetes base model for the NHS/NHSII/HPFS participants included fewer risk factors than in UKB participants because data were not available, which resulted in a lower associated C statistic (0.80 vs 0.89). Addition of sleep traits and the PGS had similar impacts on prediction model performance, with minimal changes in C statistics (Table 3). Adding sleep duration or insomnia symptoms to QDiabetes models improved specificity but at the expense of lower sensitivity, resulting in overall null improvements in model performance for sleep duration, and only a small positive total NRI improvement for insomnia symptoms (0.08 [95% CI 0.01, 0.16]). When both sleep duration and insomnia symptoms were added to the base QDiabetes risk model, there were no significant improvements in sensitivity, specificity or total NRI. By contrast, inclusion of the PGS led to improved sensitivity and specificity, regardless of whether sleep traits were included.

### Secondary analysis

To assess predictive ability using easy-to-measure person-reported risk factors outside clinical settings, we removed BMI, fasting blood glucose and HbA_1c_ from the prediction models. After these exclusions, the addition of sleep duration and/or insomnia symptoms to the QDiabetes model improved event discrimination minimally in UKB participants (ESM Table 3): C statistics increased from 0.7589 to 0.7608 by adding sleep duration, to 0.7593 by adding insomnia symptoms, and to 0.7609 by adding both variables. By comparison, the C statistic increased to 0.7671 after adding the PGS, and to 0.7693 by adding sleep duration, insomnia symptoms and the PGS.

As with the full QDiabetes model, when BMI and biochemical markers were removed, risk reclassification in UKB participants worsened for type 2 diabetes events, but non-event reclassification and total NRI improved with inclusion of sleep duration and insomnia symptoms. Inclusion of the PGS significantly improved event, non-event and total NRI, albeit minimally, and when sleep traits were also included, there were slightly greater or similar improvements in non-event and total NRIs: 0.16 (95% CI 0.15, 0.17) and 0.26 (95% CI 0.18, 0.33), respectively (ESM Table 3).

In NHS/NHSII/HPFS cohorts, when using models that did not include BMI and biochemical markers, there was no improvement in C statistics with inclusion of sleep duration or insomnia symptoms (ESM Table 3), and NRI changes were similar to findings in the UKB participants.

Demographic data on the 27,132 participants in the UKB cohort (6%) who had depression are presented in ESM Table 4. The discrimination of the baseline QDiabetes model for incident type 2 diabetes was high, and similar to that in the main analysis cohort (C statistic 0.9123; ESM Table 5). Models that included sleep duration, insomnia symptoms or the PGS did not show improved event discrimination (C statistics 0.9067, 0.9100 and 0.9129, respectively). However, non-event reclassification was improved by adding sleep duration (non-event NRI 0.55 [95% CI 0.18, 0.93]) or PGS (0.11 [95% CI 0.08, 0.15]) to the baseline QDiabetes model. In this subgroup with depression, inclusion of both sleep traits with or without inclusion of PGS did not improve event discrimination or total NRI.

In NHS/NHSII/HPFS participants with depression at baseline, discrimination of the QDiabetes base model improved with addition of the PGS (C statistic increase from 0.8013 to 0.8360), but not through addition of sleep duration and/or insomnia symptoms (ESM Table 5). Addition of sleep duration led to improvements in non-event NRI (0.41 [95% CI 0.06, 0.75]) and total NRI (0.21 [95% CI 0.01, 0.42]). Improvements in event discrimination (0.8608), non-event NRI (0.15 [95% CI 0.11, 0.19]) and total NRI (0.42 [95% CI 0.08–0.75]) were observed when the PGS, sleep duration and insomnia symptoms were included.

When BMI and biochemical variables were excluded from the QDiabetes models in UKB and NHS/NHSII/HPFS participants with depression, similar discrimination and reclassification patterns were observed to those observed for the whole cohort (ESM Table 6).

## Discussion

We have shown that sleep duration and insomnia symptoms are associated with incident type 2 diabetes independently of QDiabetes variables. After adjusting for QDiabetes variables, we observed a U-shaped relationship between sleep duration and type 2 diabetes risk, and a positive relationship between frequency of insomnia symptoms and type 2 diabetes risk. However, when sleep duration and insomnia symptoms were added to the QDiabetes model individually or in combination, there were no clinically relevant improvements in overall type 2 diabetes risk prediction. In contrast, there were marginal improvements when PGS data were added to QDiabetes models. Sleep duration and insomnia symptoms did not significantly improve type 2 diabetes risk prediction beyond that of the standard QDiabetes model in models that excluded BMI and biochemical markers or in people with depression at baseline. The findings in UKB participants were replicated in the NHS/NHSII/HPFS cohorts.

In low-resource settings where measurement of the key components BMI, fasting blood glucose and/or HbA_1c_ may not be feasible, and such data cannot be included in the QDiabetes tool, addition of sleep duration modestly improves discrimination between those who develop type 2 diabetes and those who do not. While it does not fully compensate for omission of these biochemical markers, sleep duration is easy to measure and its inclusion may enable more targeted and efficient HbA_1c_ and/or glucose testing for diagnostic confirmation.

### Prior studies on sleep traits

Epidemiological data and meta-analyses have demonstrated higher risk of incident type 2 diabetes in people with short and long sleep duration and insomnia [3]. These data are partially supported by a Mendelian randomisation study indicating a causal role for insomnia in type 2 diabetes, but not for sleep duration when considered as a linear continuous trait [23]. However, to the best of our knowledge, no study has assessed whether sleep duration and/or insomnia improve type 2 diabetes prediction beyond clinical variables. Subjective sleep traits can be easily assessed and, based on the available knowledge, these traits have the potential to improve type 2 diabetes prediction. However, we showed no improvement in type 2 diabetes prediction when adding sleep exposures to the QDiabetes calculator. Their predictive value was largely captured by existing QDiabetes variables, potentially largely by BMI, offering no clinical utility for the general population or in people with depression.

### Polygenic risk score

Several studies have shown that use of a type 2 diabetes PGS can improve the clinical prediction beyond that achieved using clinical variables [24–26]. However, to the best of our knowledge, this is the first study to show that inclusion of a PGS marginally improves risk prediction compared with the standard QDiabetes model, which has good performance in the UK population. The improvement was too small to suggest clear clinical benefits. However, there are properties of genetic data that could be of value. As genetic make-up is fixed at conception, use of a PGS could potentially support prediction of earlier onset of type 2 diabetes [27, 28], but the potential practical, ethical and financial challenges of implementing this approach remain substantial and require further investigation.

### Strengths

Our study had a large sample size and geographically diverse participants. It comprised high-quality data, a long follow-up period and minimal missing values. We linked UKB data with primary care and hospital data, providing greater capture of incident type 2 diabetes cases, and excluded participants with obstructive sleep apnoea due to its established links to type 2 diabetes risk and clear management pathways [29]. In addition to the C statistic, a popular measure of discrimination that usefully quantifies differences in how well models identify people at different levels of risk, we also included two additional measures of prediction: the likelihood ratio test and net reclassification. These complement the C statistic but are rarely included in prediction research. The likelihood ratio test provides statistical evidence of the difference in fit between two models, while net reclassification is clinically important as it provides a summary measure of the extent to which a new prediction model correctly improves event or non-event prediction. Finally, we replicated our analyses in three large US cohorts to assess the robustness of our findings.

### Limitations

First, although the UKB participants were recruited from a wide demographic base and geographical areas, the response rate for recruitment was only approximately 5%, introducing selection bias and limiting generalisability. While the fact that our findings were replicated in US cohorts with a considerably larger response under a different health system, these cohorts are also not representative of the general population. There is evidence of a ‘healthy volunteer’ selection bias in UKB participants, with differences in sociodemographic, physical, lifestyle and health-related characteristics compared with the general UK population [30]. Similarly the NHS/NHSII/HPFS cohorts differ from the general US population in terms of socioeconomic status, professional characteristics and health knowledge [31]. Nonetheless, our inferences of associations between sleep exposures and health outcomes are generally aligned with findings from more representative data sources [32]. Second, while primary care linkage improved coverage of diabetes diagnoses, these records were only available for approximately 45% of the UKB cohort. Third, since baseline sleep data are subjective, there is potential for misreporting. Despite this, self-reported sleep duration in UKB participants was largely concordant with objective measures obtained from activity-monitor devices and showed modest correlation with accelerometer-derived sleep duration, even though these data were collected approximately 6 years after self-reported data [33]. Fourth, we did not consider other sleep parameters such as snoring in the models. Habitual snoring is associated with an increased risk of type 2 diabetes, with sleep apnoea and obesity playing significant roles in this relationship [34, 35]. The combination of obesity and snoring amplifies this risk; individuals with a higher BMI and who snore have a significantly higher risk of developing type 2 diabetes compared with non-obese snorers and non-snorers [35]. Future studies should assess whether inclusion of additional sleep exposures improves prediction beyond the standard QDiabetes model. Fifth, while we excluded individuals with diagnosed sleep apnoea, this condition is typically underestimated [36], so some undiagnosed/undetected cases may remain in the cohorts. However, we do not anticipate that undiagnosed rates differ substantially between the UKB/NHS/NHSII/HPFS cohorts. Finally, insomnia data reflected self-reported insomnia symptoms rather than the definition of insomnia disorder described in the Diagnostic and Statistical Manual of Mental Disorders (DSM-5) [37], which requires specific duration, frequency and impairment criteria for a diagnosis. Individuals reporting insomnia symptoms in this study will reflect a wider population with sleep complaints than those with DSM-5-defined insomnia disorder. Inclusion of DSM-5-defined insomnia disorder may lead to differences in the predictive performance of the model.

Future studies may benefit from evaluating the utility of predicting type 2 diabetes using multidimensional sleep health domains (both subjective and objective), including sleep traits such as snoring, sleep regularity, timing and napping, although our previous analysis suggests that self-reported and activity monitor measures capture different characteristics [38]. As lifestyle changes can prevent type 2 diabetes or lead to remission, incorporating diet, nutrition and physical activity as an overall lifestyle score in the QDiabetes model could be explored in the future. A study of NHS and HPFS cohorts showed that adding sleep duration to a healthy lifestyle score slightly improved CVD prediction [39].

### Risk assessment tools

Diabetes risk assessment tools identify people at increased risk of diabetes. Following risk assessment, targeted interventions can be offered based on risk level [40]. People identified as low risk should be informed of their current status, but risk can increase in the future [40]. People with a moderate risk may be encouraged to modify particular risk factors for diabetes, and change their lifestyle [40]. Those at high risk may be encouraged to modify risk factors and make behavioural and lifestyle changes, or be referred to intensive lifestyle-change programmes [40].

### Conclusion

We have shown that inclusion of sleep duration and insomnia symptoms did not improve type 2 diabetes prediction beyond that achieved using QDiabetes risk variables in the general population or in people with depression. However, in resource-limited settings where biochemical markers cannot be measured, inclusion of sleep duration can modestly improve discrimination of type 2 diabetes. We also found that inclusion of a type 2 diabetes PGS improved type 2 diabetes prediction, although the improvements were too small to be of clear clinical value.

## Supplementary Information

Below is the link to the electronic supplementary material.

ESM (PDF 348 KB)

## Data Availability

The data that support the findings of this study are available from the UK Biobank. Restrictions apply to the availability of these data, which were used under license for this study. Data from the UK Biobank are available following application to the UK Biobank Access Management System (www.ukbiobank.ac.uk/register-apply). Access to the study protocol and analytical code is available from the corresponding author upon request.

## Abbreviations

HPFS: Health Professionals Follow-up Study
NHS: Nurses’ Health Study
NHSII: Nurses’ Health Study II
NRI: Net reclassification improvement
PCOS: Polycystic ovary syndrome
PGS: Polygenic risk score
UKB: UK Biobank
