# Supplementary material for: Clinical utility of self-reported sleep duration and insomnia symptoms in type 2 diabetes prediction
Source: Diabetologia. 2025 Aug 2;68(11):2523–34. doi: 10.1007/s00125-025-06503-6 (PMC12534266; doi:10.1007/s00125-025-06503-6)
Supplement: Supplementary file 1 — ESM (PDF 348 KB) [file 125_2025_6503_MOESM1_ESM.pdf]

Electronic Supplementary Material (ESM)

Clinical utility of self-reported sleep duration and insomnia symptoms in type 2 diabetes prediction

Alison K Wright, Tianyi Huang, Matthew J Carr, Arjun D Premdayal, Sushant Saluja, Hassan S Dashti, Simon G Anderson, David W Ray, Samuel E Jones, Andrew R Wood, Timothy M Frayling, Michael N Weedon, Jacqueline M Lane, Richa Saxena, Junxi Liu, Jack Bowden, Deborah A Lawlor, Susan Redline, Martin K Rutter

Table of Contents

	Page
**ESM Methods**	
**UK Biobank study participants**	2
**ESM Tables**	
**ESM Table 1.** Sex stratified comparison of type 2 diabetes prediction, assessed by C statistics and NRI, using the QDiabetes calculator with and without self-reported sleep duration and insomnia symptoms, in UKB	3
**ESM Table 2.** Ethnicity stratified comparison of type 2 diabetes prediction, assessed by C statistics and NRI, using the QDiabetes calculator with and without self-reported sleep duration and insomnia symptoms, in UKB	4-5
**ESM Table 3.** After excluding BMI and biochemical markers (fasting blood glucose, HbA1c), a comparison of type 2 diabetes prediction, assessed by C statistics and NRI, using the QDiabetes calculator with and without self-reported sleep duration and insomnia symptoms, in UKB and in validation cohorts.	6
**ESM Table 4.** Baseline characteristics of UKB participants with and without depression	7-8
**ESM Table 5.** In participants with depression, a comparison of type 2 diabetes prediction, assessed by C statistics and NRI, using the QDiabetes calculator with and without self-reported sleep duration and insomnia symptoms in UKB (n=27,132) and validation cohorts (n=28,495)	9
**ESM Table 6.** In participants with depression, and after excluding BMI and biochemical markers (fasting blood glucose, HbA1c), a comparison of type 2 diabetes prediction, assessed by C statistics and NRI, using the QDiabetes calculator with and without self-reported sleep duration and insomnia symptoms in UKB (n=27,132) and validation cohorts (n=28,495)	10

ESM Methods

UK Biobank study participants

The UK Biobank (UKB) is a large, open access, prospective study with 502,540 participants (5.5% response of those invited) who were aged 40-69 years and recruited from 22 assessment centres throughout the UK between 2006 and 2010 [1].

The consent of each participant to take part in UK Biobank remains the cornerstone of UK Biobank's activities [2]. The consent process begins with individuals receiving information about the study and being invited to an Assessment Centre. During the visit to the Assessment Centre, staff clarify the process and answer questions, and participants can then choose to sign a consent form if they wish to participate. The consent form outlines the scope of data usage and ensures participants understand their rights [3].

The UK Biobank contains a wealth of baseline data on demographics, lifestyle and environmental factors, medical history, physical clinical measures, biochemical assays, and genotyping. Linkage with national datasets is available including primary care (approximately 45% of participants resident in Scotland and Wales), hospital inpatient and outpatient episodes (Hospital Episode Statistics (HES)) and death records. All participants completed the baseline assessment comprised of a health and lifestyle questionnaire (self-completed), recording of physical and functional measurements and collection of blood, urine and saliva samples.

For the current analysis, we excluded participants with prevalent diabetes (as determined from a validated algorithm [4]), obstructive sleep apnoea and use of sleep-modifying medications at baseline assessment. In brief, the diabetes algorithm uses information on baseline medical history, diabetes medication, age and ethnicity from the self-completed and nurse interview questionnaire to assign presence and type of diabetes. Additionally, participants with missing data on self-reported sleep traits, those with type 1 diabetes diagnosed after enrolment and those who later withdrew consent were excluded. The resulting study cohort consisted of 492,114 participants.

References

1. Sudlow C, Gallacher J, Allen N, et al. UK Biobank: An Open Access Resource for Identifying the Causes of a Wide Range of Complex Diseases of Middle and Old Age. *PLOS Med* 2015;**12**(3):e1001779.
2. Basis of your participation - Privacy Notice for UK Biobank Participants. UK Biobank. 2025. Available from https://www.ukbiobank.ac.uk/explore-your-participation/basis-of-your-participation. Accessed on 18 April 2025.
3. Consent Form: UK Biobank. UK Biobank. 2006. Available from https://www.ukbiobank.ac.uk/media/05ldg1ez/consent-form-uk-biobank.pdf. Accessed on 18 April 2025.
4. Eastwood S V, Mathur R, Atkinson M, et al. Algorithms for the Capture and Adjudication of Prevalent and Incident Diabetes in UK Biobank. *PLoS One* 2016;**11**(9):e0162388.

ESM Table 1. Sex stratified comparison of type 2 diabetes prediction, assessed by C statistics and NRI, using the QDiabetes calculator with and without self-reported sleep duration and insomnia symptoms, in UKB

Model	New model compared with QDiabetes model				
	Harrell's C statistic	Net Reclassification Improvement (NRI)			Data fit p value[a]
		Event NRI	Non-event NRI	Total NRI	
MALE					
QDiabetes	0.8851	-	-	-	-
QDiabetes + sleep duration	0.8872	0.06 (-0.34, 0.46)	-0.12 (-0.533, 0.28)	-0.07 (-0.28, 0.15)	0.30
QDiabetes + insomnia symptoms	0.8845	-0.42 (-0.96, 0.13)	0.36 (-0.34, 1.06)	-0.06 (-0.39, 0.28)	0.62
QDiabetes + PGS	0.8852	0.04 (-0.04, 0.13)	0.13 (0.11, 0.14)	0.17 (0.08, 0.26)	0.003
QDiabetes + sleep duration + insomnia symptoms	0.8865	-0.04 (-0.24, 0.16)	0.03 (-0.15, 0.20)	-0.01 (-0.18, 0.15)	0.36
QDiabetes + sleep duration + insomnia symptoms + PGS	0.8866	0.03 (-0.08, 0.14)	0.13 (0.11, 0.14)	0.16 (0.05, 0.27)	0.03
FEMALE					
QDiabetes	0.9003	-	-	-	-
QDiabetes + sleep duration	0.9000	-0.22 (-0.45, 0.01)	0.36 (0.04, 0.67)	0.14 (-0.08, 0.36)	0.87
QDiabetes + insomnia symptoms	0.9008	-0.19 (-0.33, -0.04)	0.35 (0.03, 0.67)	0.16 (-0.09, 0.42)	0.74
QDiabetes + PGS	0.9018	0.08 (-0.04, 0.21)	0.14 (0.12, 0.16)	0.22 (0.10, 0.35)	<0.001
QDiabetes + sleep duration + insomnia symptoms	0.9005	0.01 (-0.18, 0.19)	0.14 (-0.08, 0.36)	0.15 (-0.04, 0.34)	0.93
QDiabetes + sleep duration+ insomnia symptoms + PGS	0.9020	0.05 (-0.10, 0.19)	0.15 (0.13, 0.17)	0.19 (0.04, 0.34)	0.08

Values for the C statistics are AUCs; values for NRI are score (95% CI)
[a]*p* values were calculated using the Cox model likelihood ratio test to assess whether significant improvements in the Cox model data fit were observed with the inclusion of sleep duration, insomnia symptoms and/or PGS compared with the standard QDiabetes model

ESM Table 2. Ethnicity stratified comparison of type 2 diabetes prediction, assessed by C statistics and NRI, using the QDiabetes calculator with and without self-reported sleep duration and insomnia symptoms, in UKB

Model	New model compared with QDiabetes model				
	Harrell's C statistic	Net Reclassification Improvement (NRI)			Data fit *p* value[a]
		Event NRI	Non-event NRI	Total NRI	
WHITE					
QDiabetes	0.8968	-	-	-	-
QDiabetes + sleep duration	0.8974	-0.17 (-0.29, -0.05)	0.36 (0.11, 0.61)	0.19 (-0.04, 0.43)	0.66
QDiabetes + insomnia symptoms	0.8962	-0.34 (-0.53, -0.15)	0.36 (0.03, 0.69)	0.02 (-0.17, 0.21)	0.57
QDiabetes + PGS	0.8976	0.05 (-0.03, 0.13)	0.14 (0.13, 0.15)	0.19 (0.11, 0.27)	<0.001
QDiabetes + sleep duration + insomnia symptoms	0.8967	-0.07 (-0.18, 0.03)	0.13 (0.01, 0.26)	0.06 (-0.05, 0.16)	0.63
QDiabetes + sleep duration + insomnia symptoms + PGS	0.8974	0.02 (-0.06, 0.09)	0.14 (0.13, 0.15)	0.16 (0.08, 0.23)	0.004
SOUTH ASIAN					
QDiabetes	0.8591	-	-	-	-
QDiabetes + sleep duration	0.8740	0.10 (-0.33, 0.52)	0.36 (0.09, 0.63)	0.46 (0.11, 0.81)	0.06
QDiabetes + insomnia symptoms	0.8748	0.29 (-0.48, 1.06)	0.06 (-0.64, 0.77)	0.35 (-0.08, 0.78)	0.24
QDiabetes + PGS	0.8642	0.03 (-0.24, 0.30)	0.04 (-0.04, 0.13)	0.08 (-0.21, 0.36)	0.55
QDiabetes + sleep duration + insomnia symptoms	0.8899	0.29 (-0.15, 0.73)	0.07 (-0.13, 0.26)	0.36 (-0.01, 0.72)	0.04
QDiabetes + sleep duration+ insomnia symptoms + PGS	0.8938	0.18 (-0.10, 0.46)	0.06 (-0.09, 0.21)	0.48 (-0.28, 1.24)	0.64

BLACK					
QDiabetes	0.9641	-	-	-	-
QDiabetes + sleep duration	0.9751	-0.33 (-1.38, 0.72)	-0.07 (-0.57, 0.43)	-0.40 (-1.39, 0.58)	1.00
QDiabetes + insomnia symptoms	0.9744	-0.11 (-0.96, 0.73)	0.59 (-0.10, 1.29)	0.48 (-0.44, 1.41)	1.00
QDiabetes + PGS	0.9665	0.33 (-0.52, 1.18)	0.09 (-0.06, 0.25)	0.42 (-0.53, 1.38)	0.08
QDiabetes + sleep duration + insomnia symptoms	0.9834	0.11 (-0.68, 0.90)	0.25 (0.02, 0.48)	0.36 (-0.43, 1.15)	0.17
QDiabetes + sleep duration+ insomnia symptoms + PGS	0.9830	0.33 (-0.48, 1.14)	0.17 (-0.01, 0.36)	0.51 (-0.32, 1.33)	0.09
MIXED, OTHER OR UNKNOWN					
QDiabetes	0.8568	-	-	-	-
QDiabetes + sleep duration	0.8720	-0.03 (-0.59, 0.52)	0.30 (-0.17, 0.76)	0.26 (-0.11, 0.64)	0.96
QDiabetes + insomnia symptoms	0.8571	0.62 (0.04, 1.20)	-0.47 (-0.88, -0.06)	0.15 (-0.17, 0.48)	0.99
QDiabetes + PGS	0.8634	0.23 (-0.12, 0.58)	0.18 (0.11, 0.24)	0.40 (0.05, 0.76)	0.78
QDiabetes + sleep duration + insomnia symptoms	0.8715	0.62 (0.14, 1.10)	-0.17 (-0.51, 0.17)	0.45 (0.08, 0.81)	0.97
QDiabetes + sleep duration+ insomnia symptoms + PGS	0.8779	0.55 (0.15, 0.96)	0.15 (0.05, 0.25)	0.70 (0.30, 1.11)	0.84

Values for the C statistics are AUCs; values for NRI are score (95% CI)

[a]*p* values were calculated using the Cox model likelihood ratio test to assess whether significant improvements in the Cox model data fit were observed with the inclusion of sleep duration, insomnia symptoms and/or PGS compared with the standard QDiabetes model

ESM Table 3. After excluding BMI and biochemical markers (fasting blood glucose, HbA1c), a comparison of type 2 diabetes prediction, assessed by C statistics and NRI, using the QDiabetes calculator with and without self-reported sleep duration and insomnia symptoms, in UKB and validation cohorts

Model	New model compared with QDiabetes model				
	Harrell's C statistic	Net Reclassification Improvement (NRI)			Data fit *p* value[a]
		Event NRI	Non-event NRI	Total NRI	
UKB cohort					
QDiabetes	0.7589	-	-	-	-
QDiabetes + sleep duration	0.7608	-0.17 (-0.25, -0.10)	0.36 (0.34, 0.37)	0.18 (0.11, 0.26)	0.01
QDiabetes + insomnia symptoms	0.7593	-0.34 (-0.47, -0.20)	0.44 (0.32, 0.56)	0.10 (0.03, 0.17)	0.21
QDiabetes + PGS	0.7671	0.12 (0.05, 0.20)	0.12 (0.11, 0.13)	0.24 (0.17, 0.32)	<0.001
QDiabetes + sleep duration + insomnia symptoms	0.7609	-0.001 (-0.11, 0.11)	0.19 (0.13, 0.24)	0.19 (0.10, 0.28)	0.01
QDiabetes + sleep duration + insomnia symptoms + PGS	0.7693	0.10 (0.02, 0.18)	0.16 (0.15, 0.17)	0.26 (0.18, 0.33)	<0.001
Validation cohorts[b]					
QDiabetes	0.7164	-	-	-	-
QDiabetes + sleep duration	0.7179	-0.21 (-0.30, -0.11)	0.31 (0.27, 0.35)	0.07 (-0.02, 0.17)	0.20
QDiabetes + insomnia symptoms	0.7174	-0.27 (-0.34, -0.20)	0.35 (0.35, 0.36)	0.08 (0.01, 0.15)	0.79
QDiabetes + PGS[c]	0.7342	0.18 (0.05, 0.30)	0.12 (0.11, 0.13)	0.30 (0.17, 0.43)	<0.001
QDiabetes + sleep duration + insomnia symptoms	0.7182	-0.09 (-0.26, 0.08)	0.15 (-0.02, 0.32)	0.06 (-0.04, 0.16)	0.32
QDiabetes + sleep duration+ insomnia symptoms + PGS[c]	0.7376	0.15 (0.001, 0.303)	0.13 (0.12, 0.15)	0.29 (0.13, 0.44)	0.004

Values for the C statistics are AUCs; values for NRI are score (95% CI)
[a]*p* values were calculated using the Cox model likelihood ratio test to assess whether significant improvements in the Cox model data fit were observed with the inclusion of sleep duration, insomnia symptoms and/or PGS compared with the standard QDiabetes model
[b]The QDiabetes model in the validation cohorts (NHS/NHSII/HPFS) did not include fasting blood glucose, HbA1c, Townsend deprivation score, diagnoses of schizophrenia, bipolar affective disorder or PCOS and use of second-generation antipsychotics
[c]Based on 35,072 NHS/NHSII/HPFS participants with genetic data

ESM Table 4. Baseline characteristics of UKB participants with and without depression

	People with Depression	People without Depression
	N=27,132<br>n (%)	N=464,982<br>n (%)
Age, years	55.4±7.8	56.6±8.1
Sex, male	9,161 (33.8)	214,285 (46.1)
Ethnicity		
White	26,026 (95.9)	438,422 (94.3)
Asian	284 (1.1)	7,496 (1.6)
Black	218 (0.8)	7,463 (1.6)
Mixed, Other or Unknown	601 (2.2)	11,601 (2.5)
Townsend deprivation score quintiles[a]		
1 (-6.26 to -3.94): least deprived	4,376 (16.1)	94,033 (20.2)
2 (-3.94 to -2.78)	4,735 (17.5)	93,478 (20.1)
3 (-2.78 to -1.33)	5,000 (18.4)	93,285 (20.1)
4 (-1.33 to 1.29)	5,609 (20.7)	92,695 (19.9)
5 (1.29 to 11.00): most deprived	7,363 (27.1)	90,934 (19.6)
Unknown	49 (0.2)	557 (0.1)
BMI, kg/m^2	28.4±5.5	27.3±4.7
missing	173 (0.6)	2,552 (0.6)
Fasting blood glucose, mmol/L (mean±SD)	5.1±1.3	5.1±1.2
missing	3,756 (13.8)	65,035 (14.0)
HbA1c, mmol/mol [%]	36±7 [5.4±0.6]	36±6 [5.4±0.6]
missing	1,715 (6.3)	31,724 (6.8)
Type 2 diabetes PGS[b]	$-4.0\times10^{-11}\pm1.00$	$-5.0\times10^{-11}\pm1.00$
missing	680 (2.5)	13,806 (3.0)
Smoking status		
Current smoker	4,578 (16.9)	47,077 (10.1)
Former smoker	6,517 (24.0)	106,560 (22.9)
Non-smoker	16,037 (59.1)	311,345 (67.0)
Family history of diabetes	6,130 (22.6)	99,771 (21.5)
Treated hypertension	6,038 (22.3)	94,765 (20.4)
History of cardiovascular disease	1,978 (7.3)	26,362 (5.7)
Schizophrenia or bipolar disorders	211 (0.8)	1,717 (0.4)
Depression	27,132 (100)	0
Gestational diabetes	78 (0.3)	705 (0.2)
Polycystic ovary syndrome	76 (0.3)	536 (0.1)
Corticosteroids	165 (0.6)	1,756 (0.4)
Second generation antipsychotics	315 (1.2)	488 (0.1)
Statins	4,874 (18.0)	74,095 (15.9)
Chronotype		
Definitely ‘morning’ person	5,238 (19.3)	113,160 (24.3)
More ‘morning’ than ‘evening’	7,530 (27.8)	147,636 (31.8)
More ‘evening than ‘morning’	8,215 (30.3)	115,885 (24.9)
Definitely ‘evening’ person	3,418 (12.6)	35,690 (7.7)

Unknown	2,731 (10.1)	52,611 (11.3)
Sleep Duration, h/day		
≤5	2,425 (8.9)	24,619 (5.3)
6	4,962 (18.3)	89,316 (19.2)
7	8,095 (29.8)	182,314 (39.2)
8	7,449 (27.5)	135,232 (29.1)
9	2,569 (9.5)	26,161 (5.6)
≥10	1,632 (6.0)	7,340 (1.6)
Insomnia symptoms		
Never/rarely	3,892 (14.3)	115,234 (24.8)
Sometimes	12,053 (44.4)	223,345 (48.0)
Usually	11,187 (41.2)	126,403 (27.2)

Values for continuous variables are means ± SD; values for categorical variables are n (%)

[a]For the Townsend deprivation score, quintile 1 indicates least deprived; quintile 5 indicates most deprived

[b]Standardised to *Z*-score. The PGS was calculated based on 110 SNPs

ESM Table 5. In participants with depression, a comparison of type 2 diabetes prediction, assessed by C statistics and NRI, using the QDiabetes calculator with and without self-reported sleep duration and insomnia symptoms in UKB (n=27,132) and validation cohorts (n=28,495)

Model	New model compared with QDiabetes model				
	Harrell's C statistic	Net Reclassification Improvement (NRI)			Data fit p value[a]
		Event NRI	Non-event NRI	Total NRI	
UKB cohort					
QDiabetes	0.9123	-	-	-	-
QDiabetes + sleep duration	0.9067	-0.36 (-0.82, 0.10)	0.55 (0.18, 0.93)	0.20 (-0.16, 0.55)	0.07
QDiabetes + insomnia symptoms	0.9100	-0.21 (-0.56, 0.15)	0.13 (-0.26, 0.51)	-0.08 (-0.45, 0.29)	0.28
QDiabetes + PGS	0.9129	0.06 (-0.25, 0.37)	0.11 (0.08, 0.15)	0.17 (-0.15, 0.48)	0.39
QDiabetes + sleep duration + insomnia symptoms	0.9045	-0.28 (-0.73, 0.17)	0.38 (0.08, 0.68)	0.10 (-0.32, 0.51)	0.52
QDiabetes + sleep duration + insomnia symptoms + PGS	0.9055	-0.06 (-0.43, 0.32)	0.17 (0.11, 0.23)	0.12 (-0.27, 0.50)	0.54
Validation cohorts[b]					
QDiabetes	0.8013	-	-	-	-
QDiabetes + sleep duration	0.8055	-0.20 (-0.53, 0.14)	0.41 (0.06, 0.75)	0.21 (0.01, 0.42)	0.11
QDiabetes + insomnia symptoms	0.8018	-0.10 (-0.65, 0.44)	0.12 (-0.44, 0.67)	0.01 (-0.16, 0.18)	0.49
QDiabetes + PGS[c]	0.8360	0.22 (-0.12, 0.56)	0.14 (0.09, 0.19)	0.36 (0.02, 0.69)	0.002
QDiabetes + sleep duration + insomnia symptoms	0.8061	-0.16 (-0.42, 0.09)	0.29 (0.03, 0.55)	0.13 (-0.11, 0.37)	0.17
QDiabetes + sleep duration + insomnia symptoms + PGS[c]	0.8608	0.26 (-0.07, 0.60)	0.15 (0.11, 0.19)	0.42 (0.08, 0.75)	0.003

Values for the C statistics are AUCs; values for NRI are score (95% CI)

[a]p values were calculated using the Cox model likelihood ratio test to assess whether significant improvements in the Cox model data fit were observed with the inclusion of sleep duration, insomnia symptoms and/or PGS compared with the standard QDiabetes model

[b]The QDiabetes model in the validation cohorts (NHS/NHSII/HPFS) did not include fasting blood glucose, HbA1c, Townsend deprivation score, diagnoses of schizophrenia, bipolar affective disorder or PCOS and use of second-generation antipsychotics

[c]Based on 5,335 NHS/NHSII/HPFS participants with genetic data (the Harrell's C statistic for the QDiabetes base model was 0.8297 in this subset)

ESM Table 6. In participants with depression, and after excluding BMI and biochemical markers (fasting blood glucose, HbA1c), a comparison of type 2 diabetes prediction, assessed by C statistics and NRI, using the QDiabetes calculator with and without self-reported sleep duration and insomnia symptoms in UKB (n=27,132) and validation cohorts (n=28,495)

Model	New model compared with QDiabetes model				
	Harrell's C statistic	Net Reclassification Improvement (NRI)			Data fit p value[a]
		Event NRI	Non-event NRI	Total NRI	
UKB cohort					
QDiabetes	0.7907	-	-	-	-
QDiabetes + sleep duration	0.7886	-0.09 (-0.42, 0.23)	0.27 (-0.02, 0.57)	0.18 (-0.15, 0.51)	0.43
QDiabetes + insomnia symptoms	0.7920	-0.21 (-0.62, 0.21)	0.18 (-0.14, 0.49)	-0.03 (-0.34, 0.26)	0.42
QDiabetes + PGS	0.7938	0.06 (-0.26, 0.38)	0.11 (0.08, 0.14)	0.16 (-0.16, 0.49)	0.11
QDiabetes + sleep duration + insomnia symptoms	0.7903	-0.17 (-0.50, 0.16)	0.31 (0.11, 0.50)	0.14 (-0.20, 0.47)	0.39
QDiabetes + sleep duration + insomnia symptoms + PGS	0.7960	-0.06 (-0.40, 0.29)	0.18 (0.12, 0.23)	0.12 (-0.25, 0.49)	0.27
Validation cohorts[b]					
QDiabetes	0.7075	-	-	-	-
QDiabetes + sleep duration	0.7173	-0.14 (-0.43, 0.15)	0.35 (0.16, 0.54)	0.21 (0.02, 0.40)	0.02
QDiabetes + insomnia symptoms	0.7072	-0.09 (-0.54, 0.36)	0.11 (-0.39, 0.61)	0.02 (-0.15, 0.18)	0.44
QDiabetes + PGS[c]	0.7724	0.22 (-0.10, 0.54)	0.13 (0.09, 0.17)	0.35 (0.03, 0.66)	0.002
QDiabetes + sleep duration + insomnia symptoms	0.7183	-0.07 (-0.31, 0.18)	0.28 (0.14, 0.42)	0.21 (-0.004, 0.428)	0.04
QDiabetes + sleep duration + insomnia symptoms + PGS[c]	0.8103	0.26 (-0.08, 0.61)	0.14 (0.10, 0.18)	0.40 (0.05, 0.76)	0.002

Values for the C statistics are AUCs; values for NRI are score (95% CI)

[a]p values were calculated using the Cox model likelihood ratio test to assess whether significant improvements in the Cox model data fit were observed with the inclusion of sleep duration, insomnia symptoms and/or PGS compared with the standard QDiabetes model

[b]The QDiabetes model in the validation cohorts (NHS/NHSII/HPFS) did not include fasting blood glucose, HbA1c, Townsend deprivation score, diagnoses of schizophrenia, bipolar affective disorder or PCOS and use of second-generation antipsychotics

[c]Based on 5,335 NHS/NHSII/HPFS participants with genetic data (the Harrell's C statistic for the QDiabetes base model was 0. 0.7708 in this subset)
